# Characterization of the complete mitochondrial genomes of two sea cucumbers, *Deima validum* and *Oneirophanta mutabilis* (Holothuroidea, Synallactida, Deimatidae): Insight into deep-sea adaptive evolution of Deimatidae

**DOI:** 10.1371/journal.pone.0323612

**Published:** 2025-05-15

**Authors:** Wendan Mu, Jun Liu, Haibin Zhang

**Affiliations:** 1 Institute of Deep-sea Science and Engineering, Chinese Academy of Sciences, Sanya, China; 2 Fourth Institute of Oceanography, Ministry of Natural Resources, Beihai, China; Chang Gung University, TAIWAN

## Abstract

The deep-sea is the largest and most extensive ecosystem on our planet with limited food availability, extreme pressure reaching hundreds of bars, perpetual darkness, frigid temperatures, and minimal oxygen levels. Mitochondria plays a key role in energy metabolism and oxygen usage, thus it may undergo adaptive evolution in response to pressures from extreme harsh environments. In this study, we present the mitochondrial genome sequences of the sea cucumbers *Deima validum* and *Oneirophanta mutabilis* collected from the South China Sea. To our knowledge, they are the first reported mitogenomes from the family Deimatidae. Similar to other sea cucumbers, both mitogenomes contain 13 PCGs, 2 rRNA genes, 22 tRNA genes (including duplication of *trnS* and *trnL*) and 1 non-coding regions. The genes in both species are distributed on the positive and negative strands, with six genes encoded on the L-strand and 31 genes encoded on the H-strand. We compared the order of genes from the 13 available holothurian mitogenomes and found a novel gene arrangement in *D. validum*. Phylogenetic analysis revealed that *D. validum* clustered with *O. mutabilis*, forming the deep-sea Deimatidae clade. The analysis of individual genes revealed the presence of three sites (90 L, 147 S, 192 V) in *nad2* and one site (28 S) in *nad5* with high posterior probabilities indicating positive selection. By comparing these features with those of shallow sea cucumbers, we predict that *nad2* and *nad5* may provide valuable insights into the molecular mechanisms at the mitochondrial level involved in Deimatidae's adaptation to the deep-sea habitat.

## 1. Introduciton

The deep sea, located below the continental shelves, constitutes the largest and most extensive ecosystem on our planet, encompassing approximately 66% of the global sea-floor area [1,2]. The organisms inhabiting this environment demonstrate remarkable survival capabilities, enduring severe conditions such as limited food availability, extreme pressure reaching hundreds of bars, perpetual darkness, frigid temperatures, and minimal oxygen levels [3].

The mitochondria, functioning as the cellular powerhouse, are responsible for generating energy through oxidative phosphorylation (OXPHOS), while their genes play crucial roles in regulating energy metabolism [4–6]. Mitochondria have their own genetic material that can replicate and transcribe independently [4]. The utilization of mitogenomes has been extensively employed in the investigation of phylogenetic relationships, genome evolution, phylogeography, and species identification [7–10]. To date, numerous marine organisms including sea urchins [11–13], sea lilies [14,15], sea star [16,17], shrimps [18,19], crabs [20,21] and shellfish [22–24] have had their complete mitochondrial genomes reported.

Most metazoan mitogenomes are circular DNA molecules, typically ranging in length from 14 to 20 kb. These mitogenomes commonly contain a total of 37 genes, including 13 protein-coding genes (PCGs), 22 transfer RNA genes (tRNAs), and two ribosomal RNA genes (rRNAs) [4,25–27]. The 13 energy pathway protein-coding genes all play crucial roles in energy metabolism and oxygen utilization [4,6], making them valuable genetic markers for investigating the molecular basis of organisms’ adaptive evolution to extreme environments [27,28]. In recent years, multiple studies have demonstrated that mitochondrial DNA may undergo positive selection in response to extreme harsh environmental pressures, such as NADH dehydrogenase genes [16,18,27,29], as well as the ATP synthase genes [16,18,27,30].

To assess the variation in mitogenome features of deep-sea sea cucumber and explore their potential molecular mechanisms for adapting to the deep-sea environment, we report the complete mitogenomes of *Deima validum* and *Oneirophanta mutabilis*, which were collected from depths of 3530 meters and 2985 meters respectively in the South China Sea. The family Deimatidae is a genuine deep-sea taxon and was established by Théel [31]. Up to now, few mitogenome data of deep-sea sea cucumbers has been reported. This study presented the complete mitogenome of the two deep-sea sea cucumbers (*D. validum* and *O. mutabilis*), and investigated their mitogenome features, codon usage, gene organization, phylogenetic relationships, and gene rearrangements. Finally, we conducted a positive selection analysis on the mitogenome of Deimatidae to gain insights into the genetic mechanisms underlying adaptive evolution in deep-sea environments within this family.

## 2. Methods

### 2.1. Sampling and DNA extraction

Specimens of *D. validum* and *O. mutabilis* were all collected by the deep-sea Human Occupied Vehicle (HOV) “*Shenhaiyongshi*” in the South China Sea. *D. validum* was collected at 3530 meters depth (18.198744, 114.354667), in September, 2017 and *O. mutabilis* at 2985 meters depth (18.269909, 113.425754), in June, 2018. The samples were dissected and the muscle tissues were preserved at –80°C in the Institute of Deep-sea Science and Engineering, CAS. The DNA was extracted from frozen preserved muscle tissues using the TIANamp Marine Animals DNA Kit (Tiangen Co. Beijing, China). All materials are collected and used in accordance with relevant agencies, national guidelines and regulations.

### 2.2. PCR amplification and sequencing

Two short fragments of the cytochrome *c* oxidase subunit 1 (*cox1*) and *12S* ribosomal RNA genes were amplified using the primers COIurF1 + COIurR2 [32] and 12S1091+12S1478 [33], respectively. The degenerate primers designed in this study were used to amplify partial sequences of *nad3*, *nad4*, and *nad5* based on conserved regions found in other sea cucumbers available from the GenBank. The complete mitogenome were amplified using species-specific primers that were designed based on the obtained sequences. Finally, the complete mitogenome of *D. validum* was amplified using four pairs of primers, while *O. mutabilis* was amplified using two pairs of primers (refer to S1 Table).

All PCR reactions were conducted using TaKaRa LA Taq polymerase on an Applied Biosystems Veriti Thermal Cycler. The PCR cycling conditions were established with an initial denaturation step at 94°C for 5 minutes, followed by 35 cycles consisting of denaturation at 94°C for 30 seconds, annealing at a temperature range of 55–62°C for 1 minute (refer to S1 Table), and extension at 72°C for a duration of 1–8 minutes depending on the anticipated length of the PCR products. The process concluded by subjecting the mixture to a final extension at 72°C for a duration of 10 minutes. The PCR samples underwent purification utilizing the SanPrep Column DNA Gel Extraction Kit (Sangon Biotech) and were subjected to bidirectional sequencing using the PCR primers on an ABI 3730x1 DNA Analyzer (Applied Biosystems Inc.).

### 2.3. Sequence analysis and gene annotation

The raw sequences underwent processing using Phred with a quality score of 20 and were subsequently assembled in Phrap utilizing default parameters [34,35]. Then, all sequence quality and assemblies underwent manual verification in Consed [36]. The BLAST (http://blast.ncbi.nlm.nih.gov/Blast.cgi) and ORFfinder (http://www.ncbi.nlm.nih.gov/projects/gorf/orfig.cgi) were utilized for the identification of rRNA genes and PCGs. The tRNA genes and their secondary structures were identified using the MITOS webserver program (http://mitos.bioinf.uni-leipzig.de/index.py) [37] and tRNAscan-SE 2.0 (http://lowelab.ucsc.edu/tRNAscan-SE/) [38]. The estimation of codon usage analysis was conducted using MEGA v5.0 [39]. The mitochondrial gene map was generated using GenomeVx [40]. The AT skew = [A-T] / [A + T] and GC skew = [G-C] / [G + C] were employed to characterize the variation in base composition within the class Holothuroidea [41]. CREx [42] was used to conduct pair-wise comparison of the mtDNA gene orders to determine rearrangement events.

### 2.4. Phylogenetic analysis

The phylogenetic relationships were assessed by conducting a phylogenetic analysis using the complete mitogenomes of fifteen echinoderms, including the two obtained in this study. The mtDNA sequences utilized are documented in S2 Table. *Strongylocentrotus purpuratus* (Echinoidea) and *Paracentrotus lividus* were designated as the outgroup. A set of concatenated amino acid sequences from thirteen partitioned PCGs were aligned using MAFFT v7.037b [43]. The removal of poorly aligned regions was achieved through the application of Gblocks v0.91b [44]. The Bayesian Inference (BI) and Maximum Likelihood (ML) were performed using the evolutionary substitution model recommended by ProtTest v3.4 [45], which is listed in S3 Table. The Bayesian analysis was performed using MrBayes v3.2.6 [46], with the best fit models applied to each partition (see S3 Table). The analyses were conducted with two parallel runs, each with three hot and one cold chain, for a total of 5,000,000 generations (sampling was performed every 100 generations). Convergence of independent runs was verified using Tracer 1.7 [47]. After discarding the initial 25,000 trees as a “burn in” phase, we employed the remaining 25,000 sampled trees to derive estimates for both the consensus tree with a majority rule of 50% and the Bayesian posterior probabilities (PP). The software MEGA 5.0 [39] was employed to conduct maximum likelihood (ML) analysis, utilizing the most appropriate models for each partition and performing 1000 replicates.

### 2.5. Positive selection analysis

Comparing the ratio of nonsynonymous to synonymous substitutions (*ω* = *dN/dS*) has emerged as a valuable approach for quantifying the influence of natural selection on adaptive evolution [48]. The values of *ω* indicate changes in selection pressure, with *ω* > 1 indicating positive selection, *ω* = 1 indicating neutrality, and *ω* < 1 indicating purifying selection [49]. The assessment of selective pressure on the mitogenome of sea cucumbers was conducted using the CODEML program from the pamlX package [50,51]. All the models accurately adjusted the average nucleotide frequencies across the three codon positions (Codon-Freq = 2). The combined dataset of 13 PCGs utilized the “one-ratios” model (model 0), “free-ratios” model (model 1), and “two-ratios” model to demonstrate variations in selective pressure between *Deima validum* and *Oneirophanta mutabilis*, compared to the other nine shallow sea sea cucumbers. The two branch-site models (A and A-null) were employed to examine whether these genes have experienced positive selection at specific amino acid sites. The Bayesian posterior probabilities of the specific selected sites were calculated using Bayes Empirical Bayes (BEB) [52] analysis.

## 3. Results and discussion

### 3.1. Mitogenome content and gene organization

**Fig 1:**
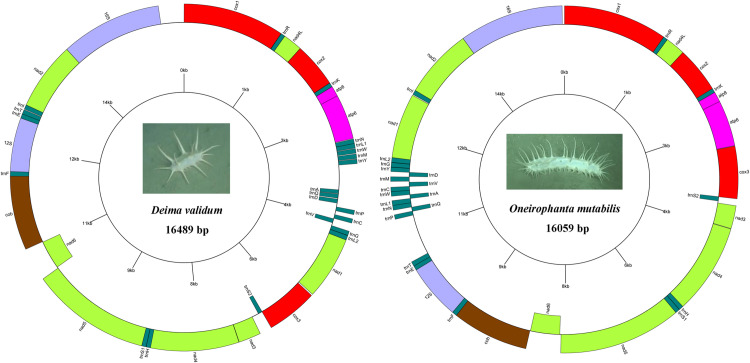
Mitochondrial gene map of *Deima validum* and *Oneirophanta mutabilis.* All of 37 genes are encoded on the both strands. Genes for proteins and rRNAs are shown with standard abbreviation. Genes for tRNAs are designated by a single letter for the corresponding amino acid with two leucine tRNAs and two serine tRNAs differentiated by numerals.

**Fig 2:**
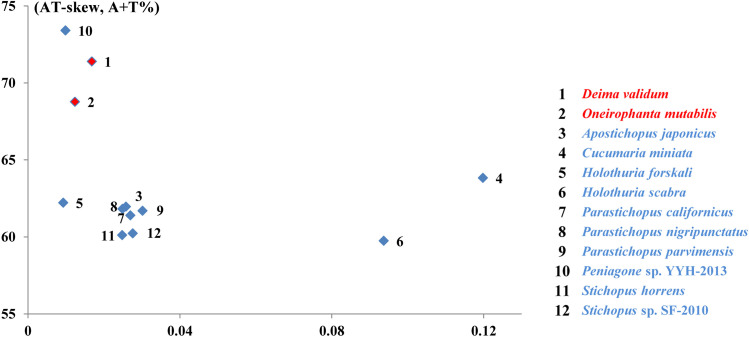
A + T% vs AT skew (a) in the 12 sea cucumber mitochondrial genomes. Values are calculated on the positive strand for the full length of the mitogenomes. The X-axis provides the skew values, while the Y axis provides the A + T values.

The complete mtDNA sequences of *D. validum* and *O. mutabili* are 16,489 bp and 16,059 in size, respectively, and their structural organization are depicted in Fig 1, S4 and S5 Tables. Both mitogenomes contain 13 PCGs, 2 rRNA, 22 tRNA (including duplication of *trnS* and *trnL*) and 1 non-coding regions. The genes in both species are distributed on the positive and negative strands, with six genes encoded on the L-strand and 31 genes encoded on the H-strand. The AT contents of mitochondrial genomes of *D. validum* and *O. mutabilis* were 71.40% and 68.78%, respectively. Both of them were higher than those of the nine known shallow-sea sea cucumber mitochondrial genomes (Fig 2, Table 1). According to Bohlin et al. [53], the relative allocation of resources towards nucleotides and amino acids can be inferred from the AT content. Chen et al. [54] found that A + T/U base pairs offer greater cost-effectiveness than G + C pairs, leading to lower energy expenditure on nucleotide synthesis in low-GC genomes when compared to high-GC genomes.

**Table 1:** Genomic characteristics of Holothuroidea mtDNAs.

Species	Accession number	Genome		Protein-coding genes		*16S* gene		*12S* gene		tRNAs		Non-coding regions		Reference
		Length (bp)	A + T (%)	Length (bp)	A + T (%)	Length (bp)	A + T (%)	Length (bp)	A + T (%)	Length (bp)	A + T (%)	Length (bp)	A + T (%)	
*Deima validum*	MK617315	16489	71.40	11385	71.06	1552	72.23	827	67.23	1503	69.00	1252	79.31	This study
*Oneirophanta mutabilis*	MK617318	16059	68.78	11388	68.10	1558	71.18	825	66.79	1503	69.06	826	75.42	This study
*Apostichopus japonicus*	NC_012616	16099	61.96	11387	61.45	1562	62.48	826	61.74	1512	62.30	915	66.89	Sun et al. [57]
*Holothuria forskali*	NC_013884	15841	62.22	11365	62.13	1577	61.45	830	61.20	1440	60.62	577	71.75	Perseke et al. [58]
*Holothuria scabra*	NC_027086	15779	59.74	11360	59.68	1532	60.77	820	57.44	1515	58.35	566	65.72	Xia et al. [59]
*Cucumaria miniata*	NC_005929	17538	63.83	11339	62.25	1319	64.90	881	65.27	1505	62.25	2500	69.08	Scouras et al. [60]
*Apostichopus nigripunctatus*	NC_013432	16112	61.82	11378	61.39	1564	61.70	826	61.62	1527	62.15	848	68.16	
*Apostichopus californicus*	NC_026727	16727	61.40	11385	60.61	1564	62.21	824	61.89	1526	62.39	1466	65.83	
*Apostichopus parvimensis*	NC_029699	16120	61.69	11360	61.21	1606	62.52	828	61.96	1521	61.93	860	66.63	
*Peniagone* sp. YYH-2013	KF915304	15507	73.41	11346	72.59	1461	73.85	830	71.08	1412	76.77	547	85.19	
*Stichopus horrens*	NC_014454	16257	60.11	11397	59.82	1642	61.02	820	58.41	1520	62.70	903	59.91	Fan et al. [61]
*Stichopus* sp. SF-2010	NC_014452	16257	60.23	11384	59.80	1653	61.65	810	59.51	1531	62.12	902	60.98	

AT-skew and A + T content of the positive strand are shown in Fig 2 for the two species in this study and other 10 sea cucumbers. The AT-skew displayed significant variation across different species, ranging from 0.0093 (5: *Holothuria forskali*) to 0.11980 (4: *Cucumaria miniate*), with the mitogenomes of *D. validum* and *O. mutabilis* showing a strong bias towards A over T (AT-skew = 0.01681, 0.01236 respectively) (Fig 2). The two mitogenomes exhibit different gene arrangements for both PCGs and tRNA genes. All the mitochondrial DNA sequences have been submitted to the GenBank under accession numbers *D. validum* **MK617315** and *O. mutabilis* **MK617318.**

### 3.2. Protein-coding genes

The mitogenomes of both *D. validum* and *O. Mutabilis* consist of 13 PCGs (*cox1*-*cox3*, *atp6*, *atp8*, *nad4L*, *nad1*-*nad6*, *cob*). All the PCGs were located on the positive strand, except for *nad6* which was encoded on the negative strand. These features have been consistently observed in all published holothurian mitogenomes. Thirteen PCGs of *D. validum* and *O. Mutabilis* exhibit the typical feature in metazoan mitogenomes by initiating with the start codon ATG [26]. The majority of PCGs in *D. validum* and *O. Mutabilis* end with the stop codon TAA, while a small number of genes terminate with TAG (S6 Table). The combined length of the PCGs sequences in *D. validum* and *O. Mutabilis* is 11385 bp (with an A + T% content of 71.06) and 11388 bp (with an A + T% content of 68.10), respectively (as shown in Table 1) (Table 1). The total number of codons (except stop codon) in *D. validum* and *O. Mutabilis* are 3782 and 3783, respectively.

**Fig 3:**
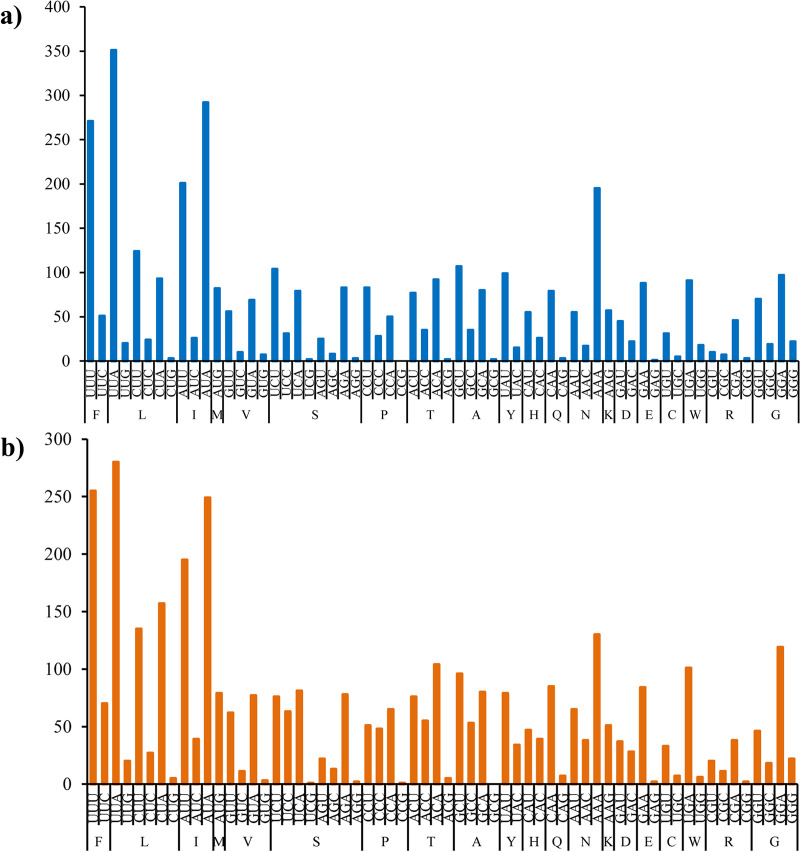
Codon usage in *Deima validum* (a) and *Oneirophanta mutabilis* (b). All codons for amino acids have been classified. Each amino acid is designated by a single letter for the corresponding codon. X-axis and y-axis represent the used times of each codon.

The codon usage of *D. validum* and *O. Mutabilis* is shown in Fig 3. Among the two sea cucumbers in this study, leucine (L) is the most frequently used amino acid, while cysteine (C) is the least frequently used. The A + T content of the third codon position in *D. validum* and *O. Mutabilis* (84.56%, 79.90%) is evidently higher than that of the first (63.93%, 58.29%) and second positions (64.44%, 63.21%). This occurrence has been recorded in various research studies, which encompass starfish, scallops, abalone, and oysters as well [16,24,55,56].

### 3.3. tRNA genes and rRNA genes

The identification of tRNA genes in *D. validum* and *O. Mutabilis* was accomplished by analyzing their potential secondary structures through the utilization of the MITOS webserver [37] and the tRNAscan-SE 2.0 [38] (refer to S1 and S2 Figs). There were two *trnS* and two *trnL* in both *D. validum* and *O. mutabilis*, which were distinguished by their different anticodons sequences. Among the tRNA genes identified in *D. validum*, twenty-one exhibited a conserved clover-leaf secondary structure, while one lacked a DHU arm from *trnS1*. Also in *O. mutabilis*, only one gene *trnI* absented a DHU arm. Loss of the DHU arm has also been observed in other Loss of the DHU arm has also been observed in other sea cucumbers, such as *Benthodytes marianensis* [29].

Like most metazoan mitogenomes, both *D. validum* and *O. mutabilis* contained two ribosomal genes (*12S* and *16S*) (Table 1). The boundaries of the *16S* and *12S* genes were established by aligning them with other sea cucumber mitogenomes. The *12S* sequences of *D. validum* and *O. mutabilis* are 827 bp (with an A + T% content of 67.23) and 825 bp (with an A + T% content of 66.79), respectively. The length of the *16S* sequences in *D. validum* and *O. mutabilis* is 1552 bp (with an A + T% content of 72.23) and 1558 bp (with an A + T% content of 71.18), respectively. These lengths are commonly observed in Holothuroidea, while the AT contents exhibit higher values compared to the other nine shallow-sea sea cucumbers (Table 1).

### 3.4. Phylogenetic analysis

**Fig 4:**
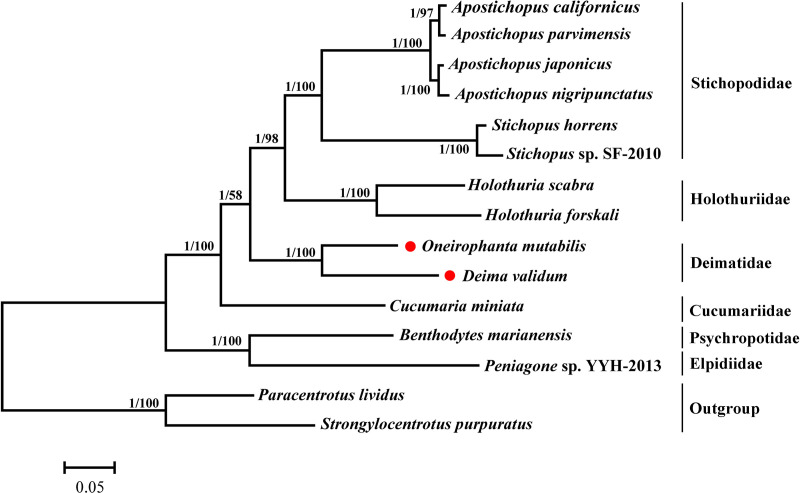
Phylogenetic trees based on the concatenated amino acid of 13 protein-coding genes. The branch lengths are determined with ML analysis. The *Paracentrotus lividus* and *Strongylocentrotus purpuratus* are used as outgroup. In BI and ML trees, the first number at each node is Bayesian posterior probability and the second number is the bootstrap probability of ML analyses. The red dot highlights the species sequenced in this study.

To explore the evolutionary relationships among the 13 Holothuroidea species, we constructed phylogenetic trees using the amino acid sequences of concatenated mitochondrial PCGs (Fig 4). The tree topologies obtained from both Bayesian Inference (BI) and Maximum Likelihood (ML) analyses were identical (Fig 4). The phylogenetic analyses revealed a strong clustering of *D. validum* with *O. mutabilis* (bootstrap support = 100 / posterior probabilities = 1) in the Deimatidae clade. It revealed that Elpidiidae and Psychropotidae was positioned at the base of the (Cucumariidae + (Deimatidae + Holothuriidae + Stichopodidae)) clade. The taxonomic relationships among Deimatidae have undergone changes throughout the years. This group was first recognized at the taxonomic rank of order by Théel 1882. Smirnov [62,63] removed the group of Deimatidae from Elasipodida to Aspidochirotida on the basis of ossicle morphology. Miller et al. [64] established the order of Synallactida by constructing phylogenetic trees based on mitochondrial genes, ribosomal RNA and nuclear genes, and recovered Deimatidae into this order. However, the availability of complete mitogenomes for Deimatidae species remains limited. Further investigation into the diverse Deimatidae species is imperative to enhance our comprehension of their evolutionary relationships.

### 3.5. Gene rearrangements

**Fig 5:**
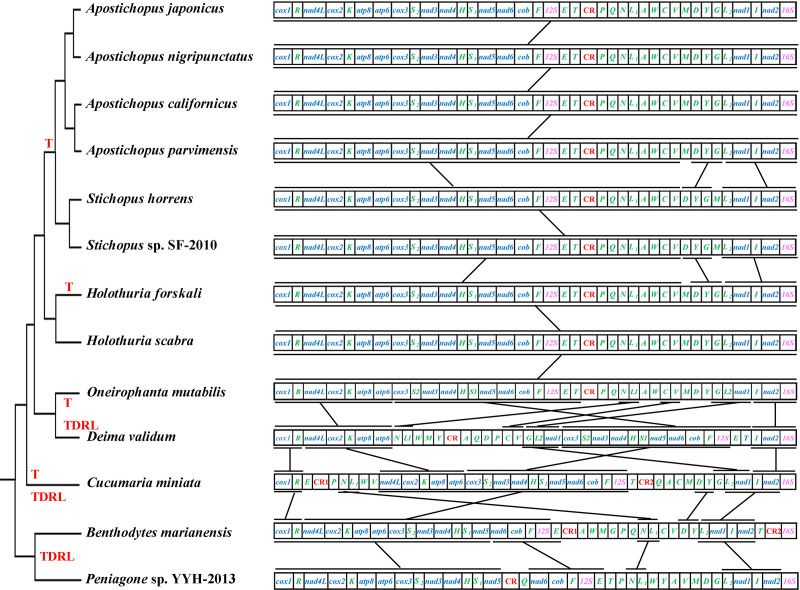
Comparison of mitochondrial gene arrangement in Holothuroidea. For the purpose of presentation, the circular mitogenomes are linearized at the boundary between *cox1* and *16S*. Genes and control regions are shown as boxes. Control regions are abbreviated as CR and are highlighted in red color. tRNA genes are named with their single letter amino acid abbreviations. Gene segments are not draw to scale.

**Fig 6:**
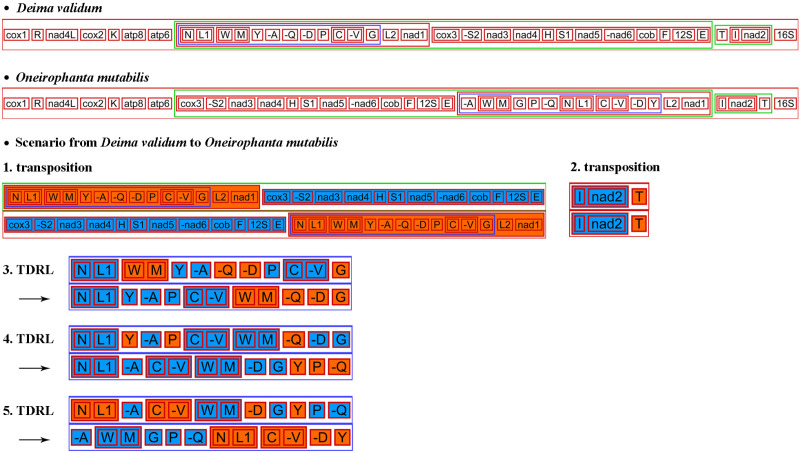
Evolution of gene order in mitochondrial genomes Deimatidae explained by CREx.

The comparison of mitochondrial gene arrangements may serve as a valuable approach for conducting phylogenetic studies [65,66]. Over the past twenty-five years, numerous studies have been conducted on mitochondrial gene orders in Holothuroidea [29,58,67,68]. Mitochondrial genome rearrangement involves four mechanisms: tandem duplication random losses (TDRLs), transposition, inversion (reversals), and reverse transposition [67]. In this study, the side by side comparison of the phylogenetic tree and gene orders of *D. validum* and *O. mutabilis* with the other 11 holothuroid species reveal the pattern of mitochondrial genome evolution (Fig 5). The CREx analysis indicated that TDRL and transposition may have been involved in the evolution of mitochondrial genomes in holothuroids (Fig 5). Except for *D. validum*, the gene order of the 13 PCGs remains conserved among the remaining 12 holothuroid species, with only a few tRNA genes exhibiting rearrangements. The location of *nad1* gene in *D. validum* has been translocated. The mitochondrial gene order of *Apostichopus japonicas*, *Apostichopus nigripunctatus*, *Apostichopus californicus*, *Apostichopus paruimensis*, *Holothuria forskali*, *Holothuria scabra* and *Oneirophanta mutabilis* is completely identical to each other. Compared with the previous seven sea cucumbers, a rearrangement of one tRNA gene (*trnM*) has been observed in *Stichopus horrens* and *Stichopus* sp. SF-2010. The species *D. validum* and *O. mutabilis* share a presence of six identical gene blocks as well as four small blocks (Fig 5). Through the analysis of software CREx, two gene transposition and three TDRL events occurred between *D. validum* and *O. mutabilis* (Fig 6). The species *D. validum* and *C. miniata* share a total of five identical gene blocks, along with three smaller blocks (Fig 5). Gene transposition (T) and tandem duplication random losses (TDRLs) events also occured in *D. validum* and *C. miniata*. By comparing *D. validum* with *C. miniata*, *Peniagone* sp.YYH-2013, and *B. marianensis*, the presence of rearranged tRNA genes can be identified. These findings support previous reports suggesting that tRNA genes are highly mobile elements within the mitochondrial genome [61,69]. This study show that the mitochondrial gene order is relatively conserved in shallow-sea sea cucumbers. However, Our findings serve as an illustrative example demonstrating that, within a taxonomic group characterized by a conserved mitochondrial gene order, rearrangements can occur in taxa inhabiting extreme deep-sea environments. Consequently, the gene arrangement of *D. validum* mitochondrial genome stands out among the published gene arrangements of sea cucumbers.

### 3.6. Positive selection analysis

The colonization of the deep sea could potentially influence mitochondrial gene function, prompting us to explore possible selective pressures within Deimatidae. The results of the selective pressure analyses are presented in Table 2. Comparing the family Deimatidae with nine other shallow-sea sea cucumbers using the two-ratio model, no significant difference was found in their *ω* ratios when we set the family Deimatidae as a foreground branch (*p* > 0.05). This suggests that the *ω* ratio of the Deimatidae branch (*ω*1 = 0.04777) is not significantly different from that of the other nine shallow-sea sea cucumbers (*ω*0 = 0.05304). However, The analysis of individual genes revealed the presence of three sites (90 L, 147 S, 192 V) in *nad2* and one site (28 S) in *nad5* with high posterior probabilities indicating positive selection (Table 2).

**Table 2:** Selective pressure analyses of the mitochondrial genes of Deimatidae.

	Branch model										
**Trees**	**Model**	**lnL**		**Estimates of parameters**			**Model compared**		**2ΔlnL**		**LRT *p*-value**
ML/BI	Model 1	-73761.45165					Model 1 versus Model 0		412.49084		0.00000
	Two ratio	-73967.54991		ω0 = 0.05304 ω1 = 0.04777			Two ratio versus Model 0		0.29432		0.58748
	Model 0	-73967.69707		ω = 0.05269							
	**Branch site model (BSM)**										
**Gene**	**Model**	**lnL**	**Estimates of parameters**					**Model compared**	**2ΔlnL**	**LRT *p*-value**	**Positive sites**
*nad2*	Model A	-6898.46338	site class	0	1	2a	2b	Model A versus Model A null	6.47168	0.01096	90 L 0.992^**^
			proportion	0.82116	0.09175	0.07834	0.00875				147 S 0.962^*^
			Background ω	0.04275	1.00000	0.04275	1.00000				192 V 0.987^*^
			Foreground ω	0.04275	1.00000	17.62769	17.62769				
	Model A null	-6901.69922									
*nad5*	Model A	-12284.20162	site class	0	1	2a	2b	Model A versus Model A null	4.11768	0.04244	28 S 0.996^**^
			proportion	0.76240	0.21822	0.01506	0.00431				
			Background ω	0.04192	1.00000	0.04192	1.00000				
			Foreground ω	0.04192	1.00000	10.81961	10.81961				
	Model A null	-12286.26046									

ω = d_N_/d_S_; Model 0: “one-ratios” model; Model 1: “free-ratios” model; Two-ratio: “two-ratios” model.

**posterior probability > 99%;

*posterior probability > 95%.

Similar findings have been observed in other deep-sea organisms, such as sea stars and sea anemones, leading to the conclusion that this phenomenon may be attributed to their adaptive evolution in response to the environment [16,27]. Survival under the extreme and harsh environment of the deep sea may necessitate an adapted energy metabolism [3,70]. The NADH dehydrogenase complex, functioning as a proton pump [71,72], serves as the initial and largest enzyme complex in the respiratory chain, potentially affecting metabolic efficiency [73]. Consequently, any mutations occurring within these subunits have the potential to impede the efficiency of the proton-pumping process. The NADH dehydrogenase complex in mammalian mitogenomes exhibits evidence of adaptive evolution, playing crucial roles in facilitating environmental adaptation among mammals [72]. Previous studies have identified *nad2* and *nad5* as pivotal determinants in the adaptive evolution of alvinocaridid shrimp and starfish mitogenomes [16,18].Therefore, the identification of positive selection in *nad2* and *nad5* within this study provides valuable insights into the molecular mechanisms at the mitochondrial level involved in Deimatidae's adaptation to the deep-sea habitat.

## 4. Conclusions

The present study characterized the complete mitochondrial genomes of the deep-sea species *D. validum* and *O. mutabilis.* The mitogenomes of both *D. validum* and *O. mutabili* consist of 13 PCGs, 22 tRNA (including duplications of *trnL* and *trnS*), 2 rRNA, and 1 non-coding region. The genes in both species are distributed on the positive and negative strands, with six genes encoded on the L-strand and 31 genes encoded on the H-strand. We conducted a comprehensive analysis of the mitogenome content, codon usage, gene organization, phylogenetic relationships, gene rearrangement, and positive selection in sea cucumbers *D. validum* and *O. mutabili*. Moreover, the gene arrangement of *D. validum* mitochondrial genome is unique among the published gene arrangements of sea cucumbers. The present study represents the first investigation into the mitogenomes of a deep-sea member that belongs to the Deimatidae family, providing valuable insights into how Deimatidae species adapt to the challenging deep-sea environment.

## Supporting information

S1 Table Primers used for amplifying and sequencing the mitogenome of *Deima validum* and *Oneirophanta mutabilis.* (DOCX)

S2 Table List of taxa used in the phylogenetic analysis. (DOCX)

S3 Table The information of alignment length and amino acid substitution models applied to each partition gene. (DOCX)

S4 Table Gene content of the *Deima validum* mitogenome. (DOCX)

S5 Table Gene content of the *Oneirophanta mutabilis* mitogenome. (DOCX)

S6 Table The length, start codon and stop codon of the protein-coding genes of sea cucumber. (DOCX)

S1 Fig Putative secondary structures for the 22 transfer RNAs of the *Deima validum* mitogenome. (JPG)

S2 Fig Putative secondary structures for the 22 transfer RNAs of the *Oneirophanta mutabilis* mitogenome. (JPG)
