# Supplementary material for: Characterization of the complete mitochondrial genomes of two sea cucumbers, Deima validum and Oneirophanta mutabilis (Holothuroidea, Synallactida, Deimatidae): Insight into deep-sea adaptive evolution of Deimatidae
Source: PLoS One. 2025 May 15;20(5):e0323612. doi: 10.1371/journal.pone.0323612 (PMC12080781; doi:10.1371/journal.pone.0323612)
Supplement: S1 Table — (DOCX) [file pone.0323612.s001.docx]

**Supplementary Table 1: Primers used for amplifying and sequencing the mitogenome of *Deima validum* and *Oneirophanta mutabilis*.**

| Species name | Name | Sequence(5’-3’) | Region | Annealing temperature | Location | Reference |
| --- | --- | --- | --- | --- | --- | --- |
| *D. validum* | COIurF1 | ACTGCCCACGCCCTAGTAATGATATTTTTTATGGTRATGCC | *cox1* | 62℃ | 175-215 | Petrov et al. [33] |
|  | COIurR2 | TCGTGTGTCTACGTCCATTCCTACTGTRAACATRTG | *cox1* |  | 871-906 |  |
|  | 12S1091 | AAAAAGCTTCAAACTGGGATTAGATACCCCACTAT | *12S* | 55℃ | 12815-12849 | Kocher et al. [34] |
|  | 12S1478 | TGACTGCAGAGGGTGACGGGCGGTGTGT | *12S* |  | 13207-13234 |  |
|  | S1-F | CCCAACGTAAAGCTATAACTCC | *cox1* | 55℃ | 1433-1545 | In this study |
|  | S1-R | GTGAAATCCATAAATAGGCATC | *nad4* |  | 8005-8026 |  |
|  | S2-F | AAAATCTGCACGTCTTCC | *nad3* | 55℃ | 7183-7200 | In this study |
|  | S2-R | ACAGTCTTTACGGGCATT | *nad5* |  | 10756-10773 |  |
|  | S3-F | TAAAATAGAACGCCTACAAGCT | *nad4* | 55℃ | 7804-7825 | In this study |
|  | S3-R | TAGAGGTGAGCTGACGACGATG | *12S* |  | 13003-13024 |  |
|  | S4-F | GAGAAGTACGGCCAAACA | *12S* | 55℃ | 12875-12892 | In this study |
|  | S4-R | AAGGGATAGCAGATGAAGT | *cox1* |  | 1693-1711 |  |
| *O. mutabilis* | COIurF1 | ACTGCCCACGCCCTAGTAATGATATTTTTTATGGTRATGCC | *cox1* | 62℃ | 175-215 | Petrov et al. [33] |
|  | COIurR2 | TCGTGTGTCTACGTCCATTCCTACTGTRAACATRTG | *cox1* |  | 871-906 |  |
|  | 12S1091 | AAAAAGCTTCAAACTGGGATTAGATACCCCACTAT | *12S* | 55℃ | 10717-10205 | Kocher et al. [34] |
|  | 12S1478 | TGACTGCAGAGGGTGACGGGCGGTGTGT | *12S* |  | 10564-10591 |  |
|  | Y1-F | GAACAGGATGAACCCTCTACCC | *cox1* | 55℃ | 368-389 | In this study |
|  | Y1-R | GAGCTGCACCTTGATCTGACGT | *12S* |  | 10414-10435 |  |
|  | Y2-F | TACGTCAGATCAAGGTGCAG | *12S* | 55℃ | 10413-10432 | In this study |
|  | Y2-R | TAGGGGAGGGTAGAGGGTTC | *cox1* |  | 377-396 |  |
