# Supplementary material for: Characterization of the complete mitochondrial genomes of two sea cucumbers, Deima validum and Oneirophanta mutabilis (Holothuroidea, Synallactida, Deimatidae): Insight into deep-sea adaptive evolution of Deimatidae
Source: PLoS One. 2025 May 15;20(5):e0323612. doi: 10.1371/journal.pone.0323612 (PMC12080781; doi:10.1371/journal.pone.0323612)
Supplement: S2 Table — (DOCX) [file pone.0323612.s002.docx]

**Supplementary Table 2: List of taxa used in the phylogenetic analysis.**

| **Species name** | **Classification** | **Accession Number** | **Reference** |
| --- | --- | --- | --- |
| **Echinoidea** |  |  |  |
| *Strongylocentrotus purpuratus* | Echinoidea; Echinoida; Strongylocentrotidae; Strongylocentrotus | NC_001453 | Qureshi et al. [12] |
| *Paracentrotus lividus* | Echinoidea; Echinoida; Echinidae; Paracentrotus | NC_001572 | Cantatore et al. [13] |
|  |  |  |  |
| **Holothuroidea** |  |  |  |
| *Deima validum* | Holothuroidea; Synallactida; Deimatidae; Deima | MK617315 | This study |
| *Oneirophanta mutabilis* | Holothuroidea; Synallactida; Deimatidae; Oneirophanta | MK617318 | This study |
| *Benthodytes marianensis* | Holothuroidea; Elasipodida; Psychropotidae; Benthodytes | MH208310 | Mu et al. [30] |
| *Holothuria forskali* | Holothuroidea; Holothuriida; Holothuriidae; Holothuria | NC_013884 | Perseke et al. [59] |
| *Holothuria scabra* | Holothuroidea; Holothuriida; Holothuriidae; Holothuria | NC_027086 | Xia et al. [60] |
| *Cucumaria miniata* | Holothuroidea; Dendrochirotida; Cucumariidae; Cucumaria | NC_005929 | Scouras et al. [61] |
| *Apostichopus japonicus* | Holothuroidea; Synallactida; Stichopodidae; Apostichopus | NC_012616 | Sun et al. [58] |
| *Apostichopus nigripunctatus* | Holothuroidea; Synallactida; Stichopodidae; Apostichopus | NC_013432 |  |
| *Apostichopus californicus* | Holothuroidea; Synallactida; Stichopodidae; Apostichopus | NC_026727 |  |
| *Apostichopus parvimensis* | Holothuroidea; Synallactida; Stichopodidae; Apostichopus | NC_029699 |  |
| *Peniagone* sp. YYH-2013 | Holothuroidea; Elasipodida; Elpidiidae; Peniagone | KF915304 |  |
| *Stichopus horrens* | Holothuroidea; Synallactida; Stichopodidae; Stichopus | NC_014454 | Fan et al. [62] |
| *Stichopus* sp. SF-2010 | Holothuroidea; Synallactida; Stichopodidae; Stichopus | NC_014452 |  |
