# Supplementary material for: Characterization of the complete mitochondrial genomes of two sea cucumbers, Deima validum and Oneirophanta mutabilis (Holothuroidea, Synallactida, Deimatidae): Insight into deep-sea adaptive evolution of Deimatidae
Source: PLoS One. 2025 May 15;20(5):e0323612. doi: 10.1371/journal.pone.0323612 (PMC12080781; doi:10.1371/journal.pone.0323612)
Supplement: S3 Table — (DOCX) [file pone.0323612.s003.docx]

**Supplementary Table 3: The information of alignment length and amino acid substitution models applied to each partition gene.**

| Partition | Alignment length | Substitution models |
| --- | --- | --- |
| *cox1* | 517 | MtREV+I+G |
| *cox2* | 229 | MtREV+G |
| *cox3* | 260 | MtArt+G |
| *cob* | 376 | MtArt+G |
| *nad1* | 317 | MtArt+G |
| *nad2* | 341 | MtArt+G+F |
| *nad3* | 106 | MtREV+I+G |
| *nad4* | 422 | CpREV+G+F |
| *nad4L* | 97 | MtMam+G |
| *nad5* | 589 | MtREV+I+G+F |
| *nad6* | 162 | MtArt+G |
| *atp6* | 221 | MtArt+G+F |
| *atp8* | 59 | MtArt+I+G+F |
