# Supplementary material for: Characterization of the complete mitochondrial genomes of two sea cucumbers, Deima validum and Oneirophanta mutabilis (Holothuroidea, Synallactida, Deimatidae): Insight into deep-sea adaptive evolution of Deimatidae
Source: PLoS One. 2025 May 15;20(5):e0323612. doi: 10.1371/journal.pone.0323612 (PMC12080781; doi:10.1371/journal.pone.0323612)
Supplement: S4 Table — (DOCX) [file pone.0323612.s004.docx]

**Supplementary Table 4: Gene content of the *Deima validum* mitogenome**

| Gene | Location | | Size | | Codon | | Intergenic  Nucleotide (bp) | Strand |
| --- | --- | --- | --- | --- | --- | --- | --- | --- |
|  | Start | End | Nucleotide (bp) | Amino acid | Start | Stop |  |  |
| *cox1* | 1 | 1554 | 1554 | 517 | ATG | TAA | 0 | + |
| *trnR* | 1559 | 1624 | 66 |  |  |  | 4 | + |
| *nad4L* | 1625 | 1921 | 297 | 98 | ATG | TAA | 0 | + |
| *cox2* | 1922 | 2611 | 690 | 229 | ATG | TAA | 0 | + |
| *trnK* | 2613 | 2677 | 65 |  |  |  | 1 | + |
| *atp8* | 2678 | 2848 | 171 | 56 | ATG | TAA | 0 | + |
| *atp6* | 2836 | 3525 | 690 | 229 | ATG | TAA | -13 | + |
| *trnN* | 3535 | 3603 | 69 |  |  |  | 9 | + |
| *trnL1* | 3604 | 3675 | 72 |  |  |  | 0 | + |
| *trnW* | 3684 | 3752 | 69 |  |  |  | 8 | + |
| *trnM* | 3765 | 3835 | 71 |  |  |  | 12 | + |
| *trnY* | 3844 | 3911 | 68 |  |  |  | 8 | + |
| *trnA* | 4331 | 4395 | 65 |  |  |  | 419 | - |
| *trnQ* | 4404 | 4473 | 70 |  |  |  | 8 | - |
| *trnD* | 4490 | 4557 | 68 |  |  |  | 16 | - |
| *trnP* | 4623 | 4688 | 66 |  |  |  | 65 | + |
| *trnC* | 4756 | 4820 | 65 |  |  |  | 67 | + |
| *trnV* | 4830 | 4899 | 70 |  |  |  | 9 | - |
| *trnG* | 4949 | 5015 | 67 |  |  |  | 49 | + |
| *trnL2* | 5018 | 5088 | 71 |  |  |  | 2 | + |
| *nad1* | 5089 | 6060 | 972 | 323 | ATG | TAG | 0 | + |
| *cox3* | 6082 | 6864 | 783 | 260 | ATG | TAA | 21 | + |
| *trnS_2_* | 6863 | 6933 | 71 |  |  |  | -2 | - |
| *nad3* | 7052 | 7396 | 345 | 114 | ATG | TAA | 118 | + |
| *nad4* | 7400 | 8764 | 1365 | 454 | ATG | TAG | 3 | + |
| *trnH* | 8755 | 8821 | 67 |  |  |  | -10 | + |
| *trnS_1_* | 8823 | 8890 | 68 |  |  |  | 1 | + |
| *nad5* | 8891 | 10729 | 1839 | 612 | ATG | TAA | 0 | + |
| *nad6* | 10752 | 11240 | 489 | 162 | ATG | TAA | 22 | - |
| *cob* | 11249 | 12391 | 1143 | 380 | ATG | TAA | 8 | + |
| *trnF* | 12387 | 12457 | 71 |  |  |  | -5 | + |
| *12S* | 12458 | 13284 | 827 |  |  |  | 0 | + |
| *trnE* | 13285 | 13351 | 67 |  |  |  | 0 | + |
| *trnT* | 13352 | 13420 | 69 |  |  |  | 0 | + |
| *trnI* | 13431 | 13498 | 68 |  |  |  | 10 | + |
| *nad2* | 13499 | 14545 | 1047 | 348 | ATG | TAA | 0 | + |
| *16S* | 14546 | 16097 | 1552 |  |  |  | 0 | + |
