# Supplementary material for: Characterization of the complete mitochondrial genomes of two sea cucumbers, Deima validum and Oneirophanta mutabilis (Holothuroidea, Synallactida, Deimatidae): Insight into deep-sea adaptive evolution of Deimatidae
Source: PLoS One. 2025 May 15;20(5):e0323612. doi: 10.1371/journal.pone.0323612 (PMC12080781; doi:10.1371/journal.pone.0323612)
Supplement: S5 Table — (DOCX) [file pone.0323612.s005.docx]

**Supplementary Table 5: Gene content of the *Oneirophanta mutabilis* mitogenome**

| Gene | Location | | Size | | Codon | | Intergenic  Nucleotide (bp) | Strand |
| --- | --- | --- | --- | --- | --- | --- | --- | --- |
|  | Start | End | Nucleotide (bp) | Amino acid | Start | Stop |  |  |
| *cox1* | 1 | 1554 | 1554 | 517 | ATG | TAA | 0 | + |
| *trnR* | 1555 | 1620 | 66 |  |  |  | 0 | + |
| *nad4L* | 1621 | 1917 | 297 | 98 | ATG | TAA | 0 | + |
| *cox2* | 1918 | 2616 | 699 | 232 | ATG | TAG | 0 | + |
| *trnK* | 2607 | 2671 | 65 |  |  |  | -10 | + |
| *atp8* | 2672 | 2836 | 165 | 54 | ATG | TAA | 0 | + |
| *atp6* | 2824 | 3513 | 690 | 229 | ATG | TAA | -13 | + |
| *cox3* | 3519 | 4301 | 783 | 260 | ATG | TAA | 5 | + |
| *trnS_2_* | 4300 | 4370 | 71 |  |  |  | -2 | - |
| *nad3* | 4405 | 4749 | 345 | 114 | ATG | TAA | 34 | + |
| *nad4* | 4755 | 6122 | 1368 | 455 | ATG | TAG | 5 | + |
| *trnH* | 6113 | 6179 | 67 |  |  |  | -10 | + |
| *trnS_1_* | 6181 | 6248 | 68 |  |  |  | 1 | + |
| *nad5* | 6249 | 8084 | 1836 | 611 | ATG | TAA | 0 | + |
| *nad6* | 8105 | 8593 | 489 | 162 | ATG | TAA | 20 | - |
| *cob* | 8602 | 9744 | 1143 | 380 | ATG | TAA | 8 | + |
| *trnF* | 9745 | 9816 | 72 |  |  |  | 0 | + |
| *12S* | 9817 | 10641 | 825 |  |  |  | 0 | + |
| *trnE* | 10642 | 10708 | 67 |  |  |  | 0 | + |
| *trnT* | 10709 | 10778 | 70 |  |  |  | 0 | + |
| *trnP* | 11452 | 11518 | 67 |  |  |  | 673 | + |
| *trnQ* | 11515 | 11584 | 70 |  |  |  | -4 | - |
| *trnN* | 11589 | 11658 | 70 |  |  |  | 4 | + |
| *trnL1* | 11659 | 11730 | 72 |  |  |  | 0 | + |
| *trnA* | 11730 | 11794 | 65 |  |  |  | -1 | - |
| *trnW* | 11796 | 11863 | 68 |  |  |  | 1 | + |
| *trnC* | 11864 | 11929 | 66 |  |  |  | 0 | + |
| *trnV* | 11929 | 11998 | 70 |  |  |  | -1 | - |
| *trnM* | 12015 | 12084 | 70 |  |  |  | 16 | + |
| *trnD* | 12093 | 12160 | 68 |  |  |  | 8 | - |
| *trnY* | 12161 | 12225 | 65 |  |  |  | 0 | + |
| *trnG* | 12228 | 12294 | 67 |  |  |  | 2 | + |
| *trnL2* | 12297 | 12367 | 71 |  |  |  | 2 | + |
| *nad1* | 12368 | 13339 | 972 | 323 | ATG | TAG | 0 | + |
| *trnI* | 13353 | 13420 | 68 |  |  |  | 13 | + |
| *nad2* | 13421 | 14467 | 1047 | 348 | ATG | TAA | 0 | + |
| *16S* | 14468 | 16025 | 1558 |  |  |  | 0 | + |
