# Supplementary material for: Characterization of the complete mitochondrial genomes of two sea cucumbers, Deima validum and Oneirophanta mutabilis (Holothuroidea, Synallactida, Deimatidae): Insight into deep-sea adaptive evolution of Deimatidae
Source: PLoS One. 2025 May 15;20(5):e0323612. doi: 10.1371/journal.pone.0323612 (PMC12080781; doi:10.1371/journal.pone.0323612)
Supplement: S6 Table — (DOCX) [file pone.0323612.s006.docx]

**Supplementary Table 6: The length, start codon and stop codon of the protein-coding genes of sea cucumber**

|  | ***D. validum*** | ***O. mutabilis*** | ***A. japonicus*** | ***H. forskali*** | ***H. scabra*** | ***C. miniata*** | ***A. nigripunctatus*** | ***A. californicus*** | ***A. parvimensis*** | ***P.* sp** | ***S. horrens*** | ***S.* sp** |
| --- | --- | --- | --- | --- | --- | --- | --- | --- | --- | --- | --- | --- |
| ***cox1*** | 1554 (ATG/TAA) | 1554 (ATG/TAA) | 1554 (ATG/TAA) | 1557 (ATG/TAA) | 1554 (ATG/TAG) | 1551 (ATG/T-) | 1554 (ATG/TAA) | 1554 (ATG/TAA) | 1554 (ATG/TAA) | 1554 (ATG/TAA) | 1554 (ATG/TAA) | 1554 (ATG/TAA) |
| ***cox2*** | 690 (ATG/TAA) | 699 (ATG/TAG) | 690 (ATG/TAA) | 688 (ATG/T-) | 688 (ATG/T-) | 690 (ATG/TAA) | 690 (ATG/TAA) | 690 (ATG/TAA) | 690 (ATG/TAA) | 685 (ATG/T-) | 690 (ATG/TAA) | 690 (ATG/TAA) |
| ***cox3*** | 783 (ATG/TAA) | 783 (ATG/TAA) | 783 (ATG/TAA) | 783 (ATG/TAA) | 783 (ATG/TAA) | 783 (ATG/TAG) | 783 (ATG/TAA) | 783 (ATG/TAA) | 783 (ATG/TAA) | 786 (ATG/TAA) | 783 (ATG/TAA) | 783 (ATG/TAA) |
| ***cob*** | 1143 (ATG/TAA) | 1143 (ATG/TAA) | 1146 (ATG/TAG) | 1143 (ATG/TAA) | 1143 (ATG/TAA) | 1141 (ATG/T-) | 1146 (ATG/TAG) | 1146 (ATG/TAG) | 1141 (ATG/T-) | 1143 (ATG/TAA) | 1143 (ATG/TAA) | 1143 (ATG/TAA) |
| ***nad1*** | 972 (ATG/TAG) | 972 (ATG/TAG) | 972 (GTG/TAA) | 972 (ATG/TAA) | 972 (ATG/TAA) | 972 (ATG/TAA) | 972 (GTG/TAA) | 972 (GTG/TAA) | 972 (ATG/TAA) | 969 (ATG/TAA) | 972 (ATG/TAA) | 972 (GTG/TAA) |
| ***nad2*** | 1047 (ATG/TAA) | 1047 (ATG/TAA) | 1044 (ATG/TAA) | 1044 (ATG/TAA) | 1044 (ATG/TAA) | 1044 (GTG/TAA) | 1044 (ATG/TAA) | 1044 (ATG/TAA) | 1044 (ATG/TAA) | 1044 (ATG/TAA) | 1047 (ATG/TAA) | 1035 (ATG/TAA) |
| ***nad3*** | 345 (ATG/TAA) | 345 (ATG/TAA) | 345 (ATG/TAA) | 345 (ATG/TAA) | 345 (ATG/TAA) | 345 (ATG/TAA) | 345 (ATG/TAA) | 345 (ATG/TAA) | 345 (ATG/TAA) | 345 (ATG/TAA) | 345 (ATG/TAA) | 345 (ATG/TAG) |
| ***nad4*** | 1365 (ATG/TAG) | 1368 (ATG/TAG) | 1371 (ATG/TAG) | 1365 (ATG/TAG) | 1357 (ATG/T-) | 1354 (ATG/T-) | 1371 (ATG/TAG) | 1371 (ATG/TAG) | 1360 (ATG/T-) | 1356 (ATG/TAG) | 1371 (ATG/TAG) | 1371 (ATG/TAG) |
| ***nad4L*** | 297 (ATG/TAA) | 297 (ATG/TAA) | 297 (ATG/TAA) | 297 (ATG/TAA) | 297 (ATG/TAA) | 297 (ATG/TAA) | 297 (ATG/TAA) | 297 (ATG/TAA) | 297 (ATG/TAA) | 297 (ATG/TAA) | 297 (ATG/TAA) | 297 (ATG/TAA) |
| ***nad5*** | 1839 (ATG/TAA) | 1836 (ATG/TAA) | 1836 (ATG/TAA) | 1833 (ATG/TAA) | 1836 (ATG/TAA) | 1824 (ATG/TAA) | 1835 (ATG/TA) | 1836 (ATG/TAA) | 1836 (ATG/TAA) | 1827 (ATG/TAA) | 1845 (ATG/TAA) | 1844 (ATG/TA) |
| ***nad6*** | 489 (ATG/TAA) | 489 (ATG/TAA) | 489 (ATG/TAG) | 489 (ATG/TAG) | 489 (ATG/TAG) | 486 (ATG/TAA) | 489 (ATG/TAG) | 489 (ATG/TAG) | 486 (GTG/TAG) | 489 (ATG/TAA) | 489 (ATG/TAA) | 489 (ATG/TAA) |
| ***atp6*** | 690 (ATG/TAA) | 690 (ATG/TAA) | 684 (ATG/TAA) | 684 (ATG/TAA) | 684 (ATG/TAA) | 684 (ATG/TAA) | 684 (ATG/TAA) | 690 (ATG/TAA) | 684 (ATG/TAA) | 684 (ATG/TAA) | 684 (ATG/TAA) | 684 (ATG/TAA) |
| ***atp8*** | 171 (ATG/TAA) | 165 (ATG/TAA) | 168 (ATG/TAA) | 165 (ATG/TAA) | 168 (ATG/TAA) | 168 (ATG/TAA) | 168 (ATG/TAA) | 168 (ATG/TAA) | 168 (ATG/TAA) | 168 (ATG/TAA) | 177 (ATG/TAA) | 177 (ATG/TAA) |
