# Supplementary figures and images for: Characterization of the complete mitochondrial genomes of two sea cucumbers, Deima validum and Oneirophanta mutabilis (Holothuroidea, Synallactida, Deimatidae): Insight into deep-sea adaptive evolution of Deimatidae
Source: PLoS One. 2025 May 15;20(5):e0323612. doi: 10.1371/journal.pone.0323612 (PMC12080781; doi:10.1371/journal.pone.0323612)

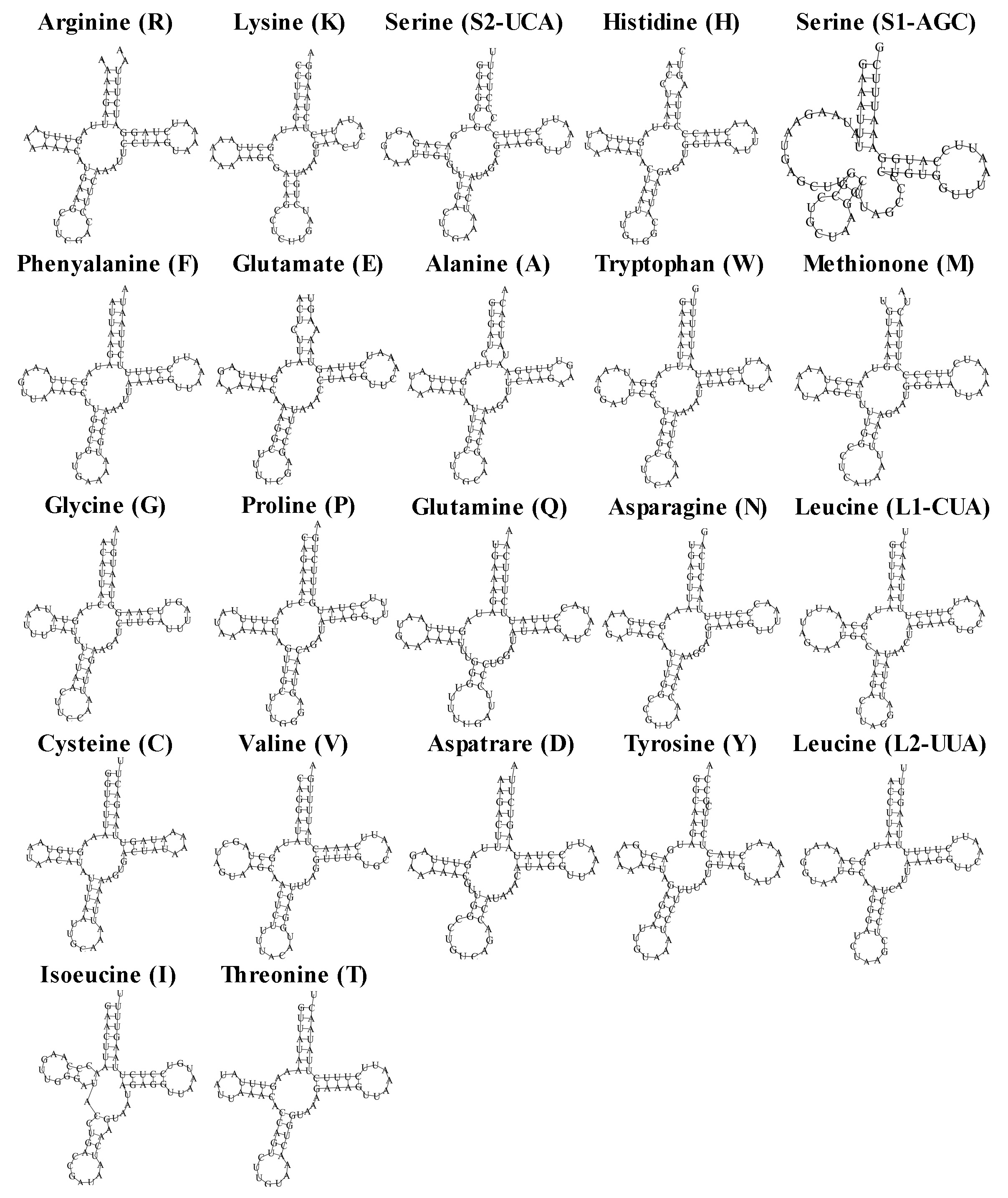

Supplement: S1 Fig — (JPG) [file pone.0323612.s007.jpg]

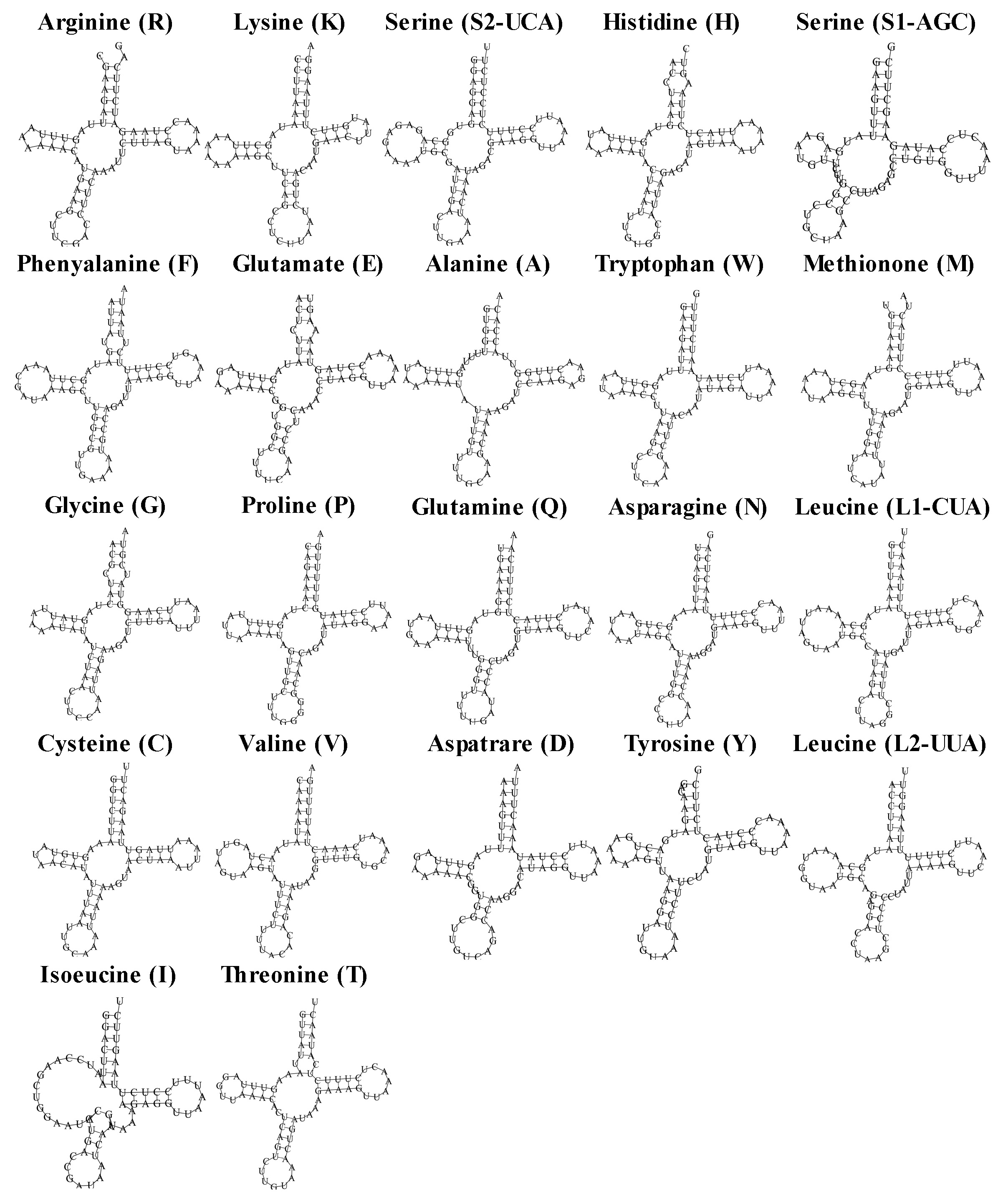

Supplement: S2 Fig — (JPG) [file pone.0323612.s008.jpg]
